# Editorial: Bipolar disorder and cognition: cognitive decline and dementia

**DOI:** 10.3389/fpsyt.2025.1567775

**Published:** 2025-02-13

**Authors:** Hirofumi Hirakawa, Hikaru Hori, Takeshi Terao

**Affiliations:** ^1^ Department of Neuropsychiatry, Oita University Faculty of Medicine, Yufu, Japan; ^2^ Department of Psychiatry, Faculty of Medicine, Fukuoka University, Fukuoka, Japan

**Keywords:** bipolar disorder, dementia, cognition, cognitive decline, mood disorder

Bipolar disorder (BD) is characterized by recurrent episodes of depression and mania/hypomania. There is scientific evidence supporting the fact that BD is often associated with cognitive decline during mood episodes and even during the euthymic state, and that cognitive decline may develop into dementia in some patients. Although our initial questions 1) Why do subjects diagnosed with bipolar disorder suffer from cognitive decline leading to dementia?, 2) Which type (e.g., Bipolar I or II) of bipolar disorder is likely to develop dementia?, 3) What factors (e.g., number of recurrences, medications such as lithium) are likely to develop or protect against dementia?, 4) What genetic factors are associated with the links between bipolar disorder, cognitive decline, and dementia?, 5) What type of dementia is likely to develop in people with bipolar disorder)? cannot be sufficiently resolved, we were able to collect the following excellent studies.

First, Matsumoto and Hamatani conducted a systematic review to investigate the influence of cognitive reserve (CR) on the relapse of bipolar episodes along with cognitive, functional, and psychopathological manifestations in BD. Their review included 36 studies and suggests that a high intelligence quotient (IQ) increases the risk of developing BD, while a high IQ is also associated with fewer bipolar episodes and protects against cognitive impairment. High levels of education and stable employment also appear to protect against cognitive impairment. In contrast, individuals with BD with low premorbid IQ, low levels of education, and unstable employment status are more likely to experience a high frequency of future bipolar episodes and/or cognitive functional decline. In conclusion, CR may be involved in preventing the relapse of bipolar episodes and in alleviating cognitive dysfunction. However, its effect on preventing the onset and relapse of bipolar episodes requires further investigation in prospective studies.

Nakamura et al. reported the case of a 74-year-old woman who experienced severe psychotic depression in old age, which led to suicide attempts during a long-term course of young-onset BD. She was finally diagnosed with dementia with Lewy bodies (DLB) based on neurocognitive symptoms and neuroimaging results. In addition, the authors reviewed recent literature on the association between BD and DLB. BD may be associated with Parkinson’s disease (PD)-related diseases, such as DLB, through dysregulation of the dopamine nervous system. A few overlapping symptoms exist between BD and DLB, making it crucial not only to differentiate between the two but also to pay attention to their comorbidities, especially in older adults. Future research is essential to identify strategies to prevent dementia in patients with BD and to develop interventions for the comorbidities of BD and DLB.

Hirakawa and Terao conducted a qualitative review of genome-wide association studies to comprehensively investigate genetic variants associated with BD and dementia. Their review included 39 studies: 20 on BD and 19 on dementia. The results showed that the calcium voltage-gated channel Alpha1C subunit (CACNA1C), gamma-aminobutyric acid B receptor 2 (GABBR2), sodium voltage-gated channel Alpha subunit 2 (SCN2A), cathepsin H (CTSH), methionine sulfoxide reductase A (MSRA), and SH3 and PX domains 2A (SH3PXD2A) genes overlapped in BD and dementia. Further genetic studies are required to comprehensively clarify the role of these genes in cognitive decline and dementia development in BD.

Oudega et al. performed a double-blind, randomized controlled study to assess the efficacy of adaptive, computerized cognitive training (CT) on executive and subjective cognitive function in patients with late-life mood disorders (LLMD). Patients over the age of 50 with partially remitted LLMD were enrolled and randomly allocated to either the CT or the active control condition (ACC) group. Over eight weeks, patients participated in 24 sessions. Thirty-eight patients were included in the study: 22 in the experimental CT group and 16 in the ACC group. The results showed no beneficial effects of an 8-week computerized CT on executive function. In conclusion, online CT, compared to ACC, did not improve executive function. However, subjective cognitive function improved in both groups, indicating that frequent cognitive training is advantageous. Future studies with more intensive training could be designed to further explore these results.

Zhang et al. conducted a cross-sectional study to investigate the relationship between childhood trauma and cognitive function in patients with BD. This study included 90 patients with BD and 94 healthy controls (HC). The results showed that childhood abuse and neglect were more prevalent in BD than in HC. Emotional abuse predicted impaired immediate memory; the number of episodes and valproate dosage were negatively correlated with cognitive function, whereas education and mood stabilizer use were positively correlated. In conclusion, the incidence of childhood trauma was higher in BD than in HC, and different types of childhood trauma had varying effects on different aspects of cognition.

Finally, Barrett et al. performed an open-label study to explore the neurocognitive effects of repeated transcranial infrared laser stimulation (TILS) in BD. Twenty-nine patients with remitted BD received six weekly TILS treatments. Cognitive test results showed that, in patients with remitted BD, TILS was effective in improving cognition, including enhanced speed and accuracy on tasks reflecting cognitive flexibility, working memory, and attentional control. Functional near-infrared spectroscopy results showed a significant reduction in the prefrontal cortex network correlations of oxygenated hemoglobin changes driven by cognitive task performance. The frontopolar cortex of the right hemisphere showed greater TILS effects than those of its left-hemisphere counterpart. In conclusion, repeated TILS is a safe intervention for improving cognition in remitted BD. To confirm TILS efficacy, a sham-controlled, double-blind randomized trial is needed.

In this Research Topic, cognitive function in BD has been linked to childhood trauma, education, and IQ. From a biological perspective, the significance of genetic factors and their associations with PD-related conditions have also been suggested. Additionally, research into novel approaches, such as computerized CT and TILS, has been conducted to enhance cognitive function in BD. Further research is needed to deepen our understanding of the mechanisms underlying cognitive decline and the onset of dementia in BD, along with preventive treatments, leading to the accumulation of scientific evidence and its application in clinical practice.

